# Differential Expression of Maternal Plasma microRNAs and Their Respective Gene Targets Can Predict Early Fetal Growth Restriction

**DOI:** 10.3390/life15020167

**Published:** 2025-01-24

**Authors:** Emmanuel Kolawole, Aparna Duggirala, Oscar Gronow, Agnieszka Lovett, Jiamiao Hu, Bee Kang Tan

**Affiliations:** 1 College of Science and Engineering, Biomedical and Clinical Science Research Centre, University of Derby, Derby DE22 1GB, UK; e.kolawole1@unimail.derby.ac.uk (E.K.); oscargronow13@gmail.com (O.G.); a.wisniewska@derby.ac.uk (A.L.); 2 Department of Cardiovascular Sciences, University of Leicester, Leicester LE1 7RH, UK; jh921@leicester.ac.uk

**Keywords:** fetal growth restriction, microRNAs, maternal blood, plasma, non-invasive diagnosis, angiogenesis, VEGF, PIGF, MMP9

## Abstract

Fetal growth restriction (FGR) is a condition where the fetus does not reach its genetically predetermined size, affecting 1 in 10 pregnancies and contributing to up to 50% of all stillbirths before 34 weeks of gestation. Current diagnostic methods primarily involve ultrasound and Doppler assessments, yet there is growing interest in identifying biomarkers for early diagnosis and improved management. This systematic review examined the role of microRNAs (miRNAs) in the pathogenesis of FGR, focusing on their potential as non-invasive biomarkers. MicroRNAs are small, non-coding RNAs that regulate gene expression. This review systematically assessed studies investigating the differential expression of miRNAs in maternal blood, serum, and plasma samples from FGR-affected pregnancies. A total of nine studies met the inclusion criteria, which showed the differential expression of a total of 48 miRNAs. miR-16-5p was consistently upregulated in multiple studies and trimesters. miR-590-3p and miR-206 were consistently upregulated in multiple trimesters. The common gene targets of these miRNAs are VEGF, PIGF, and MMP9. The downregulation of these genes contributes to impaired angiogenesis, trophoblast invasion, placental function, and fetal growth.

## 1. Introduction

Fetal growth restriction (FGR) [also known as intrauterine growth restriction (IUGR)] occurs when the fetus does not achieve its genetically predetermined potential size. FGR affects 1 in 10 pregnancies and is responsible for as high as 50% of all stillbirths occurring before 34 weeks of gestation, making it the most important risk factor for stillbirth. It is also the second leading cause of maternal pregnancy-related fetal mortality [1,2,3]. FGR is caused by factors related to the fetus, mother, or placenta. Unfortunately, delivery of the baby, either by inducing labor or Caesarean Section, remains the definitive management of mitigating the pregnancy complications associated with an FGR baby. Early diagnosis would be crucial for the effective management of the condition. Nohuz et al. have shown that detecting FGR before birth is inversely associated with the rate of resulting stillbirth [4]. 

Fetal ultrasound scans are the gold standard to assess fetal growth during pregnancy. FGR is diagnosed when the estimated fetal weight (EFW) or abdominal circumference (AC) is below the 10th percentile for gestational age, often accompanied by abnormal Doppler ultrasound findings, reduced growth velocity, and oligohydramnios, indicating compromised placental function [5]. Until the 2016 Delphi consensus, FGR was defined as a condition in which the estimated fetal weight (EFW) was less than the 10th percentile [6]. The major shortcoming of this definition, however, was its failure to differentiate between physiologically and pathologically small fetuses. The Delphi consensus provides a more sensitive definition by considering the EFW, abdominal circumference (AC), and Doppler ultrasound scan measurements such as end-diastolic flow, the pulsatility index (PI), and the cerebroplacental ratio [7]. The details on how this definition was arrived at are as follows. Consensus was undertaken to determine the cut-off values for accepted parameters. A total of 45 experts completed four rounds of the Delphi procedure. The panel was asked to rate the literature-based selected parameters for FGR on a 5-point Likert scale. Parameters with a median score of 5 and 4 on a Likert scale were considered for the second and third rounds. In the final round, possible algorithms to define early and late FGR were presented to the panel in two multiple-choice questions. For the definition of early FGR, three parameters were identified as “very important” (median score of 5): measurements of the abdominal circumference (AC), estimated fetal weight (EFW), and the pulsatility index (PI) of the UA. For the definition of late FGR, two “very important” parameters were identified: measurements of AC and EFW. FGR is typically diagnosed during the second half of pregnancy, and the difference between early and late FGR depends on whether the condition was diagnosed before or after 32 to 34 weeks of gestation [8]. In addition to the conventional diagnostic method, which typically combines the use of ultrasonic examination and Doppler velocimetry, fetal chromosomes may be also assessed for FGR-related chromosomal aberrations. Fetal samples may be obtained from the chorionic villi and amniotic fluid by invasive means. More recently, there have been attempts to identify biomarkers that may aid earlier diagnosis and timely effective management [4,9]. Previous research showed that the ratio of soluble fms-like tyrosine kinase (sFLT-1) vs. placental growth factor (PIGF), as well as the levels of soluble vascular adhesion protein-1 (sVAP-1), can predict complicated pregnancies [10]. Additionally, gene regulators, like microRNAs, are small, non-coding RNAs, with an average length of 22 nucleotides, and they can also predict complicated pregnancies. MiRNAs function as epigenetic regulators of gene expression by binding and degrading mRNAs [11]. They are also involved in intercellular signaling and could be transported from inside the cells where they are produced into the circulation [9] where they have been found to vary in their expression in various diseased conditions compared to healthy counterparts. Several studies have now explored the predictive and therapeutic potential of miRNAs in FGR [12]. The miRNAs identified to potentially have a causative role in FGR have been found and examined in the placenta, trophoblast, fetal cord blood, or maternal blood. This opens the possibility that placental protein markers together with their respective gene regulators, like microRNAs, could predict fetal growth restriction. Although several miRNAs are independently evaluated for the early diagnosis of FGR, a systematic review of uniformly regulated miRNAs and their gene targets in maternal blood of FGR pregnancies is not available to date. These circulating miRNAs could have predictive/diagnostic potential for FGR. Also, maternal blood is more easily accessible and relatively non-invasive, making microRNAs in the maternal circulation potentially useful as less invasive biomarkers in predicting/diagnosing FGR. We, therefore, sought to systematically review the role and contribution of miRNA in FGR, specifically by assessing the pattern of miRNA expression and target gene dysregulation in maternal whole blood, serum, and plasma across multiple studies and to highlight miRNAs and gene targets, which may be useful as biomarkers for the early and relatively non-invasive diagnosis of FGR. 

## 2. Materials and Methods

### 2.1. Systematic Review Registration

The record for this systematic review was registered and published on the PROSPERO register with registration number CRD42024489685. 

### 2.2. Search Strategy

To acquire pertinent studies, the following databases were searched: BioMed Central, Embase, Science Direct (Elsevier), Science Full Text Select (H.W. Wilson), Web of Science, PubMed, PubMed Central, ScienceDirect, Scopus, Springer, and Wiley Online. The search was limited to studies published in the English language. The keywords used were “fetal growth restriction” “intrauterine growth restriction” OR “FGR” OR “IUGR” AND “miRNA” AND “microRNA”.

### 2.3. Study Selection and Eligibility

Studies were screened independently by three authors (EK, AD, and BT). Conflicts were jointly resolved, and studies to be included or excluded were jointly agreed upon. This review was methodologically structured in accordance with the guidelines outlined by the Preferred Reporting Items for Systematic Reviews and Meta-Analyses (PRISMA). The studies included broadly fall into two types: cohort and case–control. The eligibility criteria included human studies and studies that investigated maternal blood/plasma/serum from SGA- or FGR-affected singleton pregnancies and assessed the differential expression of miRNAs. Also, included studies only recruited participants without apparent confounding underlying clinical conditions, such as cardiovascular diseases and diabetes, or other pregnancy complications, such as preeclampsia or gestational diabetes. Animal studies, twin studies, reviews and meta-analyses, editorials, and theses were excluded. Studies that only investigated other sample types (placenta tissue, cord blood, HUVECs, trophoblast cell lines) were excluded also.

### 2.4. Data Extraction and Quality Assessment

The following data were extracted: author, title, year of publication, journal, citation, study design, sample type, ethnicity, gestational age at sampling, maternal age, sample size, internal control, miRNAs studied, experimentally validated gene targets, assay methods, and data analysis software. The methodological quality of the included studies was assessed using the Newcastle–Ottawa scale coding system, a system designed for evaluating the quality and risk of bias in biomedical research studies. We conducted the quality assessment, and any discrepancies were resolved through consensus discussion. The scoring system provided by the Newcastle–Ottawa scale was used to interpret the quality assessment results. Table A1 in Appendix A provides a summary of the quality assessment for the studies. According to the Melnyk and Fineout–Overholt pyramid hierarchy [13] of evidence, the current systematic review belonged to the level 4 case–control and cohort-based studies.

## 3. Results

### 3.1. Search Results

**Figure 1:**
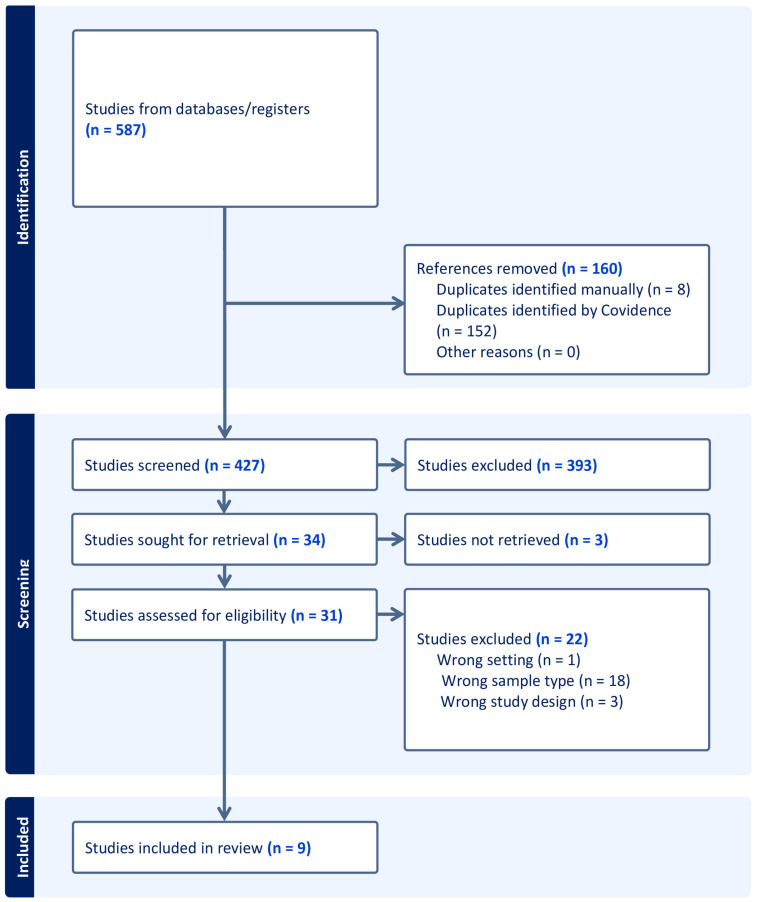
Systematic review flowchart diagram. The PRISMA flow diagram for the systematic review detailing the database searches, the number of studies screened, and studies assessed for eligibility (full-text retrieval).

The initial search was filtered to exclude editorials, newsletters, books, and articles not published in the English language and yielded 587 articles, after which 160 duplicates were removed. A total of 427 studies were screened, out of which 393 did not fit the inclusion criteria of this study. Among the thirty-four studies included, three articles’ full texts were not available; therefore, the full text from thirty-one studies was extracted. Out of the 31 studies, 22 studies were excluded because of the sample types used, e.g., placenta tissues, fetal cord blood, and trophoblast cell lines, or the wrong study design or wrong settings were adopted. The remaining nine studies met the inclusion criteria and were included in this systematic review. A graphical presentation of the step-by-step process through which the articles were selected is shown in Figure 1.

### 3.2. Study Characteristics

Nine studies investigated the expression of predetermined microRNAs in plasma (6) and whole blood (3) samples. Table 1 displays details of the characteristics of all nine studies used in the review.

### 3.3. MicroRNAs in Fetal Growth Restriction and Their Gene Targets

**Figure 2:**
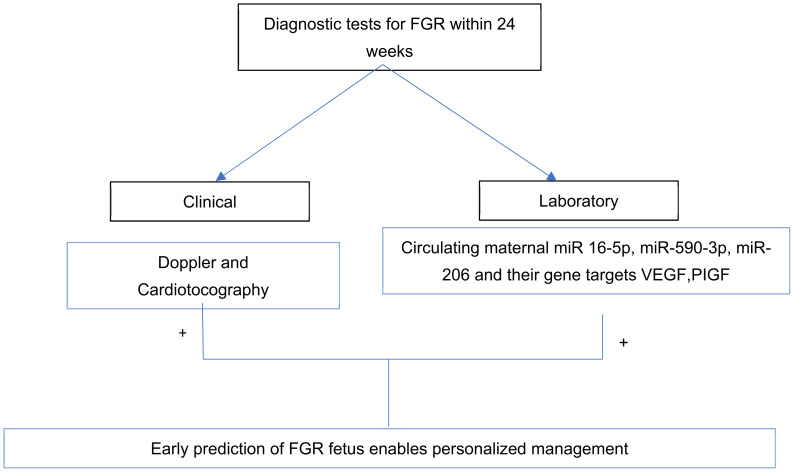
Graphical abstract showing the diagnostic potential of circulating miRNA and its gene targets in FGR.

In this systematic review, we investigated and gathered information on miRNAs that have been reported by experimental studies to be differentially expressed in maternal whole blood, serum, and plasma samples obtained from FGR-affected pregnancies. In these studies, the expression of a total of 48 miRNAs in normal and FGR-affected pregnancies was profiled. MiR-16-5p was of special interest because it was the only miRNA found to be uniformly regulated (upregulated) in two studies, in plasma and whole blood, respectively [19,21]. We searched the miRWalk database to identify experimentally validated gene targets of miR-16-5p. It was found from the search that miR-16-5p regulated angiogenesis by targeting VEGF (vascular endothelial growth factor) in endothelial progenitor cells [22]. MiR-590-3p and miR-206 were also of interest because they were upregulated in maternal plasma in multiple trimesters. Pei et al. (2021) showed the upregulation of 590-3p in the second and third trimesters [15], while Li and Liu et al. (2020) showed the upregulation of miR-206 in all three trimesters [23]. Furthermore, Li and Liu (2020) and Pei et al. (2021) experimentally identified VEGF as targets of miR-206 and miR-590-3p, respectively [23,24]. Pei et al. (2021) further identified PIGFs (placental growth factors) and MMP9 (matrix metalloproteinases) as targets of miR-590-3p [15]. VEGF is thus a central target for all three miRNAs. Table 2 represents the pattern of miR-16-5p, miR-206, and miR-590-3p regulation across trimesters in whole blood and plasma obtained from FGR-affected pregnancies. miR-16-5p was upregulated in the first and second trimesters in whole blood and plasma, respectively [19,21]. MiR-206 was upregulated all through the three trimesters [23]. MiR-590-30 was upregulated during the second and third trimesters [15], and Table 3 shows the experimentally validated gene targets of the miR-16-5p, miR-590-3p, and miR-206. Figure 2 illustrates the flowchart representation of the diagnostic potential of circulating miRNA in maternal plasma with suspected FGR cases. The other miRNAs profiled in the remaining seven studies were either non-uniformly regulated in two studies (miR-424, miR-199a, miR-146a-5p, and miR-574-3p), non-differentially expressed, or investigated only by one study. Among those miRNAs investigated by one study in different trimesters, five miRNAs showed inconsistent regulation patterns (miR-103, miR-516-5p, miR-517, miR-520a, and miR-526a). Table A2 in Appendix A provides a summarized view of all miRNAs featured in these studies and their direction of regulation.

## 4. Discussion

The objective of this systematic review was to synthesize current research on the differential expression of microRNAs in maternal blood, serum, or plasma between pregnancies complicated by fetal growth restriction (FGR) and healthy pregnancies. Our analysis incorporated findings from multiple studies, each utilizing one of the sample types mentioned and similar methodologies to identify miRNA expression patterns associated with FGR. 

The reviewed studies highlighted several miRNAs with altered expressions in FGR, though the specific miRNAs and their directions of regulation varied. Importantly, 3 miRNAs stood out from among the rest. MiR-16-5p was the only microRNA that was uniformly regulated (upregulated) in more than one study, while miR-206 and miR-590-3p were the only microRNAs uniformly regulated (upregulated) across multiple trimesters.

Many physiological changes take place in the mother’s body to accommodate and nurture the developing fetus. Vasculogenesis, the formation of new blood vessels de novo, and angiogenesis, the formation of new blood vessels from pre-existing ones, are crucial for the development of the embryo and the functioning of the placenta, respectively [24]. Suppressed placental angiogenesis and the consequent inadequate supply of oxygen and nutrients from the mother to the developing fetus is an important aspect of the pathogenesis of FGR [25]. The overexpression of miR-16-5p, miR-590-3p, and miR-206 has been shown in different studies to be involved in the emergence of various pathologies, specifically by inhibiting angiogenesis in vascular endothelial cells [26,27,28,29,30]. MiR-16-5p was shown to be upregulated in the first and third trimesters, miR-590-3p was upregulated in the second and third trimesters, and miR-206 was upregulated all through pregnancy [15,19,21,23]. Interestingly, all three miRNAs have been shown to be anti-angiogenic. They target and downregulate the VEGF mRNA by binding to its 3’ untranslated region [15,22,23]. Studies have shown that low VEGF levels in maternal serum have been shown to be characteristic of FGR during the second and third trimesters [31,32]. It is a potent factor that drives angiogenesis and vasculogenesis and stimulates the migration of hemangioblasts, precursor cells that can give rise to both endothelial and hematopoietic cells, into the blood islands (early clusters of blood cells) during embryonic development [33]. It also specifically promotes the differentiation of these hemangioblasts into endothelial cells, which are the building blocks of blood vessels [34]. Furthermore, VEGF promotes the survival of endothelial cells by inhibiting apoptosis, ensuring the maintenance of a functional vascular network [34]. Adequate levels of VEGF throughout pregnancy are necessary for a successful pregnancy and for proper fetal development [35]. These findings collectively provide evidence that low VEGF levels, resulting at least partly from its downregulation by miR-16-5p, miR-590-3p, and miR-206, contribute to the development of FGR. They also suggest that these miRNAs and VEGF may be important as potential targets for early (within the first and second trimesters) diagnosis and treatment. In vivo studies by Swanson et al. (2016) demonstrated an increase in fetal weight by employing the adenoviral delivery of VEGF in a guinea pig model of growth restriction [36]. 

The pathological upregulation of miRNA-590-3p during the second and third trimesters in maternal plasma also contributes to the development of FGR by targeting and downregulating PIGF and MMP9 in maternal plasma [15]. PIGF is a member of the VEGF family and is also classified as pro-angiogenic. In a healthy pregnancy, PIGF expression increases during the second trimester, a change that is associated with the formation of new blood vessels within the villi (finger-like projections in the placenta that facilitate the exchange of nutrients, oxygen, and waste products between maternal and fetal circulations) and the maturation and specialization of the villi for efficient nutrient and gas exchange [37]. It is one of the most studied biomarkers in FGR and is consistently insufficiently expressed all through gestation in pregnancies with placental dysfunction [16]. Zhang et al. (2019) found maternal plasma PIGF to be significantly downregulated in FGR pregnancies compared to normal [38]. In addition, MMP9 in maternal circulation is crucial for proper trophoblast invasion, placentation, and normal embryonic development. Zhu et al. (2014) found that MMP9 levels were significantly downregulated in villous tissues obtained from pregnancies affected by FGR, adversely affecting trophoblast invasion and placenta development [39]. Plaks et al. (2013) also showed morbid morphological abnormalities in MMP9-lacking embryos developing in MMP9-lacking pregnant mice [40]. The embryos were contorted, and growth was constrained. The MMP9-lacking mice also presented low serum VEGF levels, restricted trophoblast invasion, and reduced placental volume, all of which are observed in FGR. While the study by Plaks et al. was conducted using murine models, it is worthy of note as it provides evidence for the importance of MMP9 in the growth of the embryo.

In summary, from the studies primarily considered, miR-16-5p was the only miRNA that was uniformly upregulated in more than one study. MiR-590-3p and miR-206 were the only miRNAs uniformly upregulated in multiple trimesters. All other miRNAs had contrasting patterns of expression among two studies or trimesters, were examined by a single study in one trimester, or were unchanged in their expression. The three miRNAs of interest were experimentally shown to target VEGF. In addition, miR-590-3p also targeted PIGF and MMP9. 

MicroRNA-based diagnostics is an emerging molecular approach that can potentially use non-invasive body fluids to test the differentially expressed miRNAs in the maternal blood and aid clinicians in predicting the FGR in the infant. miRNAs are considered diagnostic biomarkers since they are highly stable to severe physiochemical conditions in the body fluids, which increases their potential as a diagnostic biomarker in predicting maternal and fetal health, according to Francesca et al. (2021) [41]. Next-generation sequencing technologies have been utilized to discover the novel circulating miRNA differentially expressed in mouse placental tissue. The authors showed that miRNA and mRNA are dysregulated in FGR mouse models [12]. This paper describes that the differentially expressed miRNA in the FGR mice model demonstrated enrichment of oxidative stress and apoptosis, and autophagy pathways were upregulated and angiogenesis and signal transduction pathways were downregulated. These data directly correlate to the upregulation of miR-16-5p, miR 206, and miR590-3p and thereby the downregulation of its gene targets VEGF and PLGF.

The miRNA extracted from the current systematic review belonged to targeted QPCR-based miRNA assays. Despite the stability of miRNAs in various tissue types, the diagnostic potential has been poor due to technical and experimental variations, indicating the limitation of specifying circulating miRNA as a diagnostic marker. Therefore, in this review, we extracted all the published differentially expressed miRNAs from maternal plasma from all the included studies and identified that two original research papers confirmed that miR16 is uniformly upregulated in first-trimester maternal plasma. The current systematic review highlights the need for larger cohort-based screening. The three miRNAs of interest (miR 16-5p, miR-590-3p, and miR-206) in this systematic review were experimentally shown to target VEGF. In addition, miR-590-3p also targeted PIGF and MMP9. Henceforth, future larger cohort-based studies are warranted to screen for the three miRNAs and the respective gene targets. Once the reproducibility and specificity of the tests are calculated and correlated with clinical findings, the miRNAs can be used routinely to predict the FGR in maternal blood.

### Strengths and Limitations

The strength of this systematic review is that it is the most comprehensive and up-to-date systematic review, which describes the involvement of miRNAs dysregulated in the maternal circulation and their specific experimentally verified gene targets in the context of FGR. The limitations are the heterogeneity of the studies, as well as the provision of only qualitative data by several studies, making it difficult to conduct a meta-analysis. The studies vary in internal control, measure of effect size, and trimester at sampling. More than half of the studies did not state the ethnicity of study participants. Also, only eight out of forty-eight miRNAs were investigated in more than one study, and of which only the direction of miR-16-5p regulation was consistent (except those that remained unchanged). Despite these limitations, it is still reasonable to infer the importance of miR-16-5p, miR-206, miR-590-3p, and their aforementioned gene targets as potential biomarkers for early diagnosis.

## 5. Conclusions

These findings show that the upregulation of miR-16-5p, miR-206, and miR-590-3p participates in the pathogenesis of FGR by downregulating VEGF, PIGF, and MMP9, in turn collectively altering angiogenic efficiency, apoptosis, trophoblast development and invasion, placental development, and blood flow to the placenta. Additionally, the dysregulation of the miRNAs and gene targets early in pregnancy (within the first and second trimesters) implies that they may be suitable as potential circulating biomarkers for developing a diagnostic model for the early prediction or detection of FGR. Therefore, further studies are warranted to screen for the dysregulation of miR-16-5p, miR-206, and miR-590-3p and gene targets VEGF, PIGF, and MMP9 in maternal whole blood and plasma samples through the period of pregnancy and especially across various ethnicities. We also suggest that a more thorough proteomic analysis of maternal blood, serum, and plasma from FGR-affected pregnancies be carried out to profile the dysregulation of proteins related to pregnancy and fetal development. There is scope for future in vitro and in vivo animal studies to prove the molecular pathway involving miRNA 16 and its gene target in FGR.

## Figures and Tables

**Table 1:** miRNAs of interest and their relevant information extracted from the reviewed studies.

Title	Year	Citation	Journal	Sample Type	Ethnicity	Gestational Stage at the Time of Sampling	Mean Maternal Age (FGR)	Mean Maternal Age (Control)	Number of Participants (FGR)	Number of Participants (Control)	Internal Control
MicroRNA-206 predicts raised fetal growth retardation risk through the interaction with vascular endothelial growth factor in pregnancies	2020	[14]	Medicine	Maternal plasma	Unspecified	1st trimester (≤13 weeks), 2nd trimester (14–27 weeks), 3rd trimester (≥28 weeks)	30.8	28.6	74	746	U6
MiR5903p and its targets VEGF, PIGF, and MMP9 in early, middle, and late pregnancy: their longitudinal changes and correlations with risk of fetal growth restriction	2021	[15]	Irish Journal of Medicine	Maternal plasma	Unspecified	1st trimester (week 10/11), 2nd trimester (week 20/21), 3rd trimester (week 33/34)	29	27.4	95	875	U6
Circulating MicroRNAs in Maternal Blood as Potential Biomarkers for Fetal Hypoxia In-Utero	2013	[16]	PLoS One	Maternal whole blood	Unspecified	3rd trimester (30 weeks)	31.4	30.3	12	12	RNU-48 and RNU-6b
First trimester screening of circulating C19MC microRNAs and the evaluation of their potential to predict the onset of preeclampsia and IUGR	2017	[17]	PLoS One	Maternal plasma	Caucasian	1st trimester (10–13 weeks)	34.56 (27–43)	32.71 (27–42)	18	58	cel-miR-39
Gestational hypertension, preeclampsia and intrauterine growth restriction induce dysregulation of cardiovascular and cerebrovascular disease associated microRNAs in maternal whole peripheral blood	2016	[18]	Thrombosis Research	Maternal whole blood	Caucasian		28–34.3	26.5–33	33	20	RNU38B and RNU58A
miR-16-5p, miR-103-3p, and miR-27b-3p as Early Peripheral Biomarkers of Fetal Growth Restriction	2021	[19]	Frontiers in Pediatrics	Maternal plasma	Caucasian	Within 2 weeks of hospitalization and 48 h before delivery	33.1	31.8	34	43	U6 snRNA
The levels of hypoxia-regulated microRNAs in plasma of pregnant women with fetal growth restriction	2010	[14]	Placenta	Maternal plasma	Unspecified		25.4	25.2	14	14	
Absolute and Relative Quantification of PlacentaSpecific MicroRNAs in Maternal Circulation with Placental Insufficiency–Related Complications	2012	[20]	The Journal of Molecular Diagnostics	Maternal plasma	Caucasian	15, 30, 32, and 34 weeks			11	50	miR-16 and let-7d
First-Trimester Screening for Fetal Growth Restriction and Small-for-Gestational-Age Pregnancies without Preeclampsia Using Cardiovascular Disease-Associated MicroRNA Biomarkers	2022	[21]	Biomedicines	Maternal whole peripheral blood	Caucasian	1st trimester (10–13 weeks)	FGR: 34 (22–44). SGA: 32 (23–43)	32 (25–42)	82 (5 early FGR, 77 late FG); 27 (SGA)	80 (AGA)	RNU58A and RNU38B

MiR-16-5p was an miRNA that was uniformly upregulated in more than one study. MiR-590-3p and miR-206 were uniformly upregulated in multiple trimesters. All other miRNAs had contrasting patterns of expression among two studies or trimesters, were examined by a single study and in one trimester, or were unchanged in their expression.

**Table 2:** miRNAs of interest and their relevant information extracted from the reviewed studies.

miRNA	Direction of Regulation	Citation	Sample Type	Gestational Age at Sampling	Maternal Age (FGR)	Maternal Age (Control)	Internal Control
miR-16-5p	Upregulated	[21]	Whole blood	First trimester	34 (22–44)	32 (25–42)	RNU58A and RNU38B
	Upregulated	[19]	Plasma	Third trimester	33.1	31.8	U6 snRNA
miR-206	Upregulated	[23]	Plasma	First, second, and third trimesters	30.8	28.6	U6
miR-590-3p	Upregulated	[15]	Plasma	First, second, and third trimesters	29	27.4	U6

MiR-16-5p was an miRNA that was uniformly upregulated in more than one study. MiR-590-3p and miR-206 were uniformly upregulated in multiple trimesters. All other miRNAs had contrasting patterns of expression among two studies or trimesters, were examined by a single study and in one trimester, or were unchanged in their expression.

**Table 3:** Experimentally validated gene targets for miR-16, miR-206, and miR-590-3p found in the literature under study.

miRNA	Experimentally Validated Gene Targets	Source
miR-16-5p	VEGF	[22]
miR-206	VEGF	[23]
miR-590-3p	VEGF, PIGF, MMP9	[15]

Gene targets were validated using qPCR or immunoblotting. VEGF, vascular endothelial growth factor; PIGF, placental growth factor; MMP9, matrix metalloproteinase 9.

## Data Availability

No new data were created.

## Appendix A

**Table A1:** Quality assessment of studies using the Newcastle–Ottawa scale.

Year	Citation	Selection of Study Groups				Comparability of Study Groups	Assessment of Outcome			Total
		Representativeness of Cases *	Selection of Controls *	Ascertainment of Cases by EFW + GA + Any of PI, End Diastolic Velocity Waveforms, Medical History *	Consistent Ethnicity of Cases and Controls *	Comparability of Study Groups by Controlling for Confounding Factors **	Same Internal Control for Controls and Cases *	Appropriate miRNA/Gene Expression Evaluation Method (qRT-PCR, ELISA, Immunoblotting) *	Consistent miRNA/Gene Expression Evaluation Method for Both Cases and Controls *	Total
2020	Li and Liu, 2020 [23]	*	*	*			*	*	*	6/9
2021	Pei et al., 2021 [15]	*	*				*	*	*	5/9
2013	Whitehead et al., 2013 [42]	*	*	*			*	*	*	6/9
2017	Hromadnikova et al., 2017 [17]	*	*	*	*		*	*	*	7/9
2016	Hromadnikova et al., 2016 [18]	*	*	*	*		*	*	*	7/9
2022	Hromadnikova et al., 2022 [21]	*	*		*		*	*	*	6/9
2021	Tagliaferri et al., 2021 [19]	*	*		*		*	*	*	6/9
2010	Mouillet et al., 2010 [14]	*	*					*	*	4/9
2012	Hromadnikova et al., 2012 [20]	*	*		*		*	*	*	6/9

Representativeness of cases, i.e., the cases were representative of the target population. Selection of controls, i.e., the controls were drawn from the same community as the cases. Ascertainment of cases, i.e., the paper stated that growth restriction was diagnosed using the estimated fetal weight (EFW), gestational age (GA), and at least one of the following: pulsatility index (PI), end-diastolic velocity waveforms, amniotic fluid deficiency, and medical history. Consistent ethnicity, i.e., cases and controls were said to be of the same ethnic group. Comparability of study groups by controlling for confounding factors; a single asterisk if one factor was controlled for and a double asterisk if more than one factor was controlled for.

**Table A2:** Table showing relevant information on all miRNAs extracted from the studies.

miRNA	Direction of Regulation	Citation	Sample Type	Gestational Age at Sampling	Maternal Age (FGR)	Maternal Age (Control)	Internal Control	Ethnicity
miR-424	Upregulated	[42]	Whole blood	30 weeks	31.4	30.3	RNU-48, RNU-6b	Not available
	Downregulated	[14]	Plasma		25.4	25.2		
miR-199a	Upregulated	[42]	Whole blood	30 weeks	31.4	30.3	RNU-48, RNU-6b	Not available
	Downregulated	[18]	Whole blood		28–34.3	26.5–33	RNU38B and RNU58A	Caucasian
miR-20b	Upregulated	[42]	Whole blood	30 weeks	31.4	30.3	RNU-48, RNU-6b	
miR-16	Upregulated	[21]	Whole blood	10–13 weeks	34 (22–44)	32 (25–42)	RNU58A and RNU38B	Caucasian
	Upregulated	[19]	Plasma	Within 2 weeks of hospitalization and 48 h before delivery	33.1	31.8	U6 snRNA	Caucasian
miR-146a-5p	Upregulated	[21]	Whole blood	10–13 weeks	34 (22–44)	32 (25–42)	RNU58A and RNU38B	Caucasian
	Downregulated	[18]	Whole blood		28–34.3	26.5–33	RNU38B and RNU58A	Caucasian
miR-574-3p	Upregulated	[21]	Whole blood	10–13 weeks (1st trimester)	34 (22–44)	32 (25–42)	RNU58A and RNU38B	Caucasian
	Downregulated	[18]	Whole blood		28–34.3	26.5–33	RNU38B and RNU58A	Caucasian
miR-223-3p	Unchanged	[19]	Plasma	Within 2 weeks of hospitalization and 48 h before delivery (3rd trimester)	33.1	31.8	U6 snRNA	Caucasian
miR-525	Unchanged	[17]	Plasma	10–13 weeks (1st trimester)	34.56 (27–43)	32.71 (27–42)	cel-miR-39	Caucasian
	Upregulated (between weeks 12 and 16) Normalized from week 20 to term	[20]	Plasma	15, 30, 32, and 34 weeks			C19Mc	Caucasian
miR-518b	Unchanged	[17]	Plasma	10–13 weeks (1st trimester)	34.56 (27–43)	32.71 (27–42)	cel-miR-39	Caucasian
	Unchanged	[14]	Plasma		25.4	25.2	RNU6B	Not available
	Upregulated (between weeks 12 and 16) Normalized from week 20 to term	[20]	Plasma	15, 30, 32, and 34 weeks			C19Mc	Caucasian
miR-520h	Unchanged	[17]	Plasma	10–13 weeks (1st trimester)	34.56 (27–43)	32.71 (27–42)	cel-miR-39	Caucasian
	Upregulated (between weeks 12 and 16) Normalized from week 20 to term	[20]	Plasma	15, 30, 32, and 34 weeks			C19Mc	Caucasian
miR-206	Upregulated	[23]	Plasma	≤13 weeks (1st trimester), 14–27 weeks (2nd trimester), ≥28 weeks (3rd trimester)	30.8	28.6	U6	Not available
miR-590-3p	Upregulated	[15]	Plasma	10/11 weeks (1st trimester), 20/21 weeks (2nd trimester), 33/34 (3rd trimester)	29	27.4	U6	Not available
miR-373 miR-210 miR-21	Upregulated	[42]	Whole blood	30 weeks (3rd trimester)	31.4	30.3	RNU-48 and RNU-6b	Not available
miR-20a-5p miR-145-5p miR-155-5p miR-181a-5p miR-342-3p	Upregulated	[21]	Whole blood	10–13 weeks (1st trimester)	34 (22–44)	32 (25–42)	RNU58A and RNU38B	Caucasian
miR-103	Upregulated before week 32 Downregulated between weeks 32 and 37	[19]	Plasma	Within 2 weeks of hospitalization and 48 h before delivery (3rd trimester)	33.1	31.8	U6 snRNA	Caucasian
miR-27b-3p	Upregulated	[19]	Plasma	Within 2 weeks of hospitalization and 48 h before delivery (3rd trimester)	33.1	31.8	U6 snRNA	Caucasian
miR-516-5p miR-517 * miR-520 * miR-526a	Upregulated (between weeks 12 and 16) Normalized from week 20 to term	[20]	Plasma	15, 30, 32, and 34 weeks			C19Mc	Caucasian
miR-100-5p miR-125b-5p miR-221-3p	Downregulated	[18]	Whole blood		28–34.3	26.5–33	RNU38B and RNU58A	Caucasian
miR-107	Downregulated	[19]	Plasma	Within 2 weeks of hospitalization and 48 h before delivery (3rd trimester)	33.1	31.8	U6 snRNA	Caucasian
miR-516b-5p miR-517-5p 5290a-5p	Unchanged	[17]	Plasma	10–13 weeks (1st trimester)	34.56 (27–43)	32.71 (27–42)	cel-miR-39	Caucasian
miR-143-3p miR-7a-5p miR-223-5p miR-101a-3 miR-218a-5p	Unchanged	[19]	Plasma	Within 2 weeks of hospitalization and 48 h before delivery (3rd trimester)	33.1	31.8	U6 snRNA	Caucasian
miR-27a miR-30d miR-141 miR-200c miR-205 miR-451 miR-491 miR-517a miR-518e miR-524	Unchanged	[14]	Plasma		25.4	25.2	RNU6B, RNU38B	Caucasian
miR 29c miR 409-3p miR 551a miR 454-3p miR 301a-3p	Upregulated	[43]	Serum	Third trimester	28.5 (18–36)	24(18–40)	UniSp6 RNA	Caucasian and other
miR 200b-3p miR 224-5p miR526b-5p miR28-5p miR378a-3p miR550a-3p	Downregulated	[43]	Serum	Third trimester	28.5 (18–36)	24(18–40)	UniSp6 RNA	Caucasian and other

miR-517 * is written as miRNA-517-5p. Reference [41] has mentioned as miRNA-517 *. Therefore we used the similar terminology. Similarly, miR-520 * is miRNA-520-5p.
